# Cocirculation of Hajj and non-Hajj strains among serogroup W meningococci in Italy, 2000 to 2016

**DOI:** 10.2807/1560-7917.ES.2019.24.4.1800183

**Published:** 2019-01-24

**Authors:** Cecilia Fazio, Arianna Neri, Paola Vacca, Andrea Ciammaruconi, Milena Arghittu, Anna Maria Barbui, Caterina Vocale, Paola Bernaschi, Patrizia Isola, Irene Alessandra Galanti, Antonella Mencacci, Rosella De Nittis, Maria Chironna, Anna Giammanco, Elisabetta Pagani, Alessandro Bisbano, Paola Stefanelli

**Affiliations:** 1 Department of Infectious Diseases, Istituto Superiore di Sanità, Rome, Italy; 2 Molecular Biology Section, Army Medical and Veterinary Research Center, Rome, Italy; 3 Microbiology Unit, Fondazione IRCCS Ca’ Granda, Ospedale Maggiore Policlinico, Milan, Italy; 4 Microbiology and Virology Laboratory, Molinette Hospital, Turin, Italy; 5 Unit of Clinical Microbiology, Regional Reference Centre for Microbiological Emergencies, St. Orsola Malpighi University Hospital, Bologna, Italy; 6 Microbiology Laboratory, Bambino Gesù Hospital, Rome, Italy; 7 Clinical Pathology Department, Azienda USL 6, Livorno, Italy; 8 Microbiology Laboratory, Azienda USL Toscana sud est, Arezzo, Italy; 9 Medical Microbiology Section, Dept. of Medicine, University of Perugia, Perugia, Italy; 10 Clinical Pathology Department, University Hospital, Foggia, Italy; 11 Biomedical Sciences and Human Oncology Department – Hygiene Section, University Hospital, Bari, Italy; 12 Department of Sciences for Health Promotion and Mother and Child Care “G. D’Alessandro”, University of Palermo, Palermo, Italy; 13 Microbiology and Virology Laboratory, Azienda Sanitaria dell’Alto Adige, Bolzano, Italy; 14 Epidemiology Unit ASP Crotone, Italy

**Keywords:** capsular serogroup W, clonal complex 11, invasive bacterial infections, invasive meningococcal disease, Italy, molecular methods, national surveillance system, *Neisseria meningitidis*

## Abstract

In Italy, B and C are the predominant serogroups among meningococci causing invasive diseases. Nevertheless, in the period from 2013 to 2016, an increase in serogroup W *Neisseria meningitidis* (MenW) was observed. This study intends to define the main characteristics of 63 MenW isolates responsible of invasive meningococcal disease (IMD) in Italy from 2000 to 2016. We performed whole genome sequencing on bacterial isolates or single gene sequencing on culture-negative samples to evaluate molecular heterogeneity. Our main finding was the cocirculation of the Hajj and the South American sublineages belonging to MenW/clonal complex (cc)11, which gradually surpassed the MenW/cc22 in Italy. All MenW/cc11 isolates were fully susceptible to cefotaxime, ceftriaxone, ciprofloxacin, penicillin G and rifampicin. We identified the full-length NadA protein variant 2/3, present in all the MenW/cc11. We also identified the fHbp variant 1, which we found exclusively in the MenW/cc11/Hajj sublineage. Concern about the epidemic potential of MenW/cc11 has increased worldwide since the year 2000. Continued surveillance, supported by genomic characterisation, allows high-resolution tracking of pathogen dissemination and the detection of epidemic-associated strains.

## Introduction

The history of the global spread of invasive meningococcal disease (IMD) caused by serogroup W *Neisseria meningitidis* (MenW) started in the year 2000, following an international emergency during the annual Hajj season in Saudi Arabia [1]. Before that, MenW had rarely been recorded as the cause of outbreaks but rather of sporadic IMD, with a low reported incidence [1]. Recently, MenW has been spreading in different countries worldwide [2-6]. It is of concern that in the United Kingdom (UK), MenW IMD incidence has increased year by year, reaching 24% of all IMD laboratory-confirmed cases in the epidemiological year 2014/15 [5,7]. In the Netherlands, in the epidemiological year 2015/16, the MenW incidence (0.15 cases per 100,000 inhabitants) was fivefold higher than the average incidence (0.03 cases per 100,000) reported in the period from 2002/03 to 2014/15 [7].

Whole genome sequencing (WGS) evidenced the heterogeneity of meningococci belonging to serogroup W/cc11 from different geographical areas and identified several genomic types by country [5,8]. As reported by Lucidarme et al. [5,9], genomic comparison classified most of MenW/cc11 as lineage 11.1. Moreover, this lineage includes two sublineages: Hajj and South American (previously designated the ‘South American/UK strain’) [5,9]. The first sublineage comprises the MenW/cc11 Hajj outbreak strain, the sub-Saharan African MenW/cc11 strains from epidemic periods and the endemic South African MenW/cc11 strain [9]. The second sublineage contains three main strains: the South American strain, the original UK strain (emerged in 2009 in the UK) and the UK 2013 strain [9].

The Hajj sublineage appeared in Saudi Arabia in 2000, spreading first in the African meningitis belt and then, with smaller outbreaks, in South Africa [4,8,10]. In the UK, this sublineage caused IMD in the period from 2000 to 2004; after that, it was replaced by the endogenous MenW/cc11 strain [4,9]. In France, eight MenW/cc11 cases were reported between January and April 2012 as linked to recent travel history to Sub-Saharan Africa during the MenW epidemic [11,12].

In South America, an increase in the proportion of MenW IMD cases has also been reported in early 2000 [2]. With the exception of one IMD case reported in Brazil [3], the South American MenW/cc11 isolates were not identified as Hajj strain at that time. Later, the so-called South American sublineage was responsible for clusters in southern Brazil (2003–05), in the United States (US) (2008–09) and in Chile (2010–12) [4]. In Europe, and in particular in the UK, Ireland and France, clusters of MenW belonging to the South American strain sublineage were reported more recently, 2009–15 [8]. In Sweden, the UK 2013 strain, belonging to the South American sublineage, was the cause of an increase in MenW IMD starting from 2014 [6].

In Italy, as in the other European countries, serogroups B and C are predominant, with an increase in the proportion of isolates of serogroup Y from 2% in 2007 to 17% in 2013 [13]. Even though serogroup W has rarely been identified in the country, an increase was observed following the global spread of these meningococcal strains [7,14].

Here, the genetic variation within and between meningococci associated with invasive disease was assessed by molecular analysis of *N. meningitidis* serogroup W collected from 2000 to 2016 for an overview of the phylogenetic diversity among strains circulating in Italy. Moreover, the rapid increase in MenW cases and the contemporaneous introduction of serogroup B *N. meningitidis* (MenB) vaccine (4CMenB) into the national immunisation schedule triggered us to study the vaccine antigen genes and their genetic variability. Although this vaccine is licensed for prevention of MenB disease, the antigens are not specific to this capsular group, and a potential cross-recognition and protection against other meningococcal serogroups deserves to be evaluated.

## Methods

### Surveillance of invasive meningococcal disease

The IMD National Surveillance System (NSS) is based on mandatory reporting to the Ministry of Health and to the Italian Institute of Public Health (Istituto Superiore di Sanità (ISS), http://www.iss.it/mabi). ISS, as national reference laboratory (NRL), acts as coordinator of the NSS. Within the surveillance system, the hospital laboratories collect bacterial isolates and/or clinical samples from IMD cases and send them to the NRL for serogroup identification or confirmation and for molecular investigations. The NRL collects demographic and relevant clinical data (i.e. vaccination history) from all notified IMD cases in a dedicated database. 

The data are analysed using EpiInfo software (version 3.5.3, 26 January 2011).

### Microbiological analyses

For the samples sent to the NRL, the serogroup was identified or confirmed by slide agglutination with commercial antisera (Thermo Scientific, Waltham, Massachusetts, US) or by multiplex PCR [15]. For the bacterial isolates, susceptibility to cefotaxime, ceftriaxone, ciprofloxacin, penicillin G and rifampicin was determined by the minimum inhibitory concentration (MIC) test strip method (Liofilchem, Roseto degli Abruzzi, Italy) on Mueller-Hinton agar (Thermo Scientific, Waltham, Massachusetts, US) supplemented with 5% of sheep blood. The breakpoints were those recommended by the European Committee on Antimicrobial Susceptibility Testing (EUCAST) [16]. Chromosomal DNA was extracted using the QiAmp mini kit (Qiagen, Hilden, Germany) from an overnight culture or directly from the clinical sample, blood or cerebrospinal fluid (CSF). Multilocus sequence typing (MLST), PorA and FetA typing, MenB vaccine antigen variants and *penA* gene were identified using the PubMLST.org database (http://pubmlst.org/neisseria/). The genotypic formula comprised capsular group: *porA* (P1).VR1,VR2: *fetA* VR: ST(cc). The MenW/cc11 isolates were characterised for the allelic profile of six antigen-encoding genes (*porA, porB, fetA, nadA, nhba* and *fHbp*) suggested by Mustapha et al. as typical of the main MenW/cc11 sublineages [4].

### Whole genome sequencing

Cultivated isolates were analysed by WGS. For each isolate, 1 ng of DNA was used to prepare the sequencing libraries following the Nextera XT DNA protocol. The Illumina MiSeq platform (kit v3, 600 cycles) was used for the WGS analysis. A first quality check of the raw sequence data was performed using FastQC [17]. Reads were trimmed using the software Sickle [18] to maintain a Q score > 25, and de novo assembly was carried out with the ABySS software version 1.5.2 (k parameter = 63) [19]. Contigs longer than 500 bp were selected using an ad hoc script and kept for further analysis. The final assembly ranged from 84 to 316 (median: 209) contigs per sample (N50: 10,999–59,092 bp; median: 19,790 bp), covering the ca 2.2 Mb of the *N. meningitidis* genome.

### Genome comparison

Genomes were uploaded to the PubMLST.org database (http://pubmlst.org/neisseria/) and compared using the BIGSdb Genome Comparator [20] through gene-by-gene analysis. Phylogenetic analysis of the isolates was performed by core genome MLST (cgMLST) [21]. Incomplete loci were automatically removed from the distance matrix calculation for the neighbour-net graphs. The resulting distance matrices were visualised as neighbour-net networks, generated by SplitsTree4 (version 4.13.1) [22].

### Statistics

Change in the average annual incidence of MenW from 2000 to 2012 vs 2013 to 2016 was evaluated using a negative binomial regression model.

## Results

From 2000 to 2016, 3,540 laboratory-confirmed IMD cases were reported within the NSS for IMD in Italy, with an incidence of 0.37 per 100,000 in 2016 (www.iss.it/mabi/, last access: 3 September 2018).

For 2,357 IMD cases, the capsular serogroup was identified: 1,249 were B, 861 were C, 161 were Y, 63 were W, 17 were A, five were X and one was 29E. One isolate was capsule null locus (*cnl*). The majority of cases were due to serogroups B and C, with proportions of 36% and 42%, respectively, in 2016.

**Figure 1:**
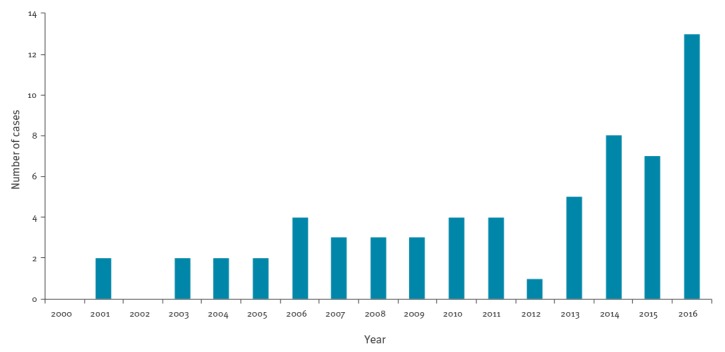
*Neisseria meningitidis* serogroup W causing invasive meningococcal disease, by year, Italy, 2000–2016 (n = 63)

As shown in Figure 1, MenW was rare from 2000 until 2012, with an average annual incidence of 0.004 per 100,000 population (30 cases). From 2013 to 2016, the average annual incidence grew to 0.01 per 100,000 population (33 cases), significantly higher than in the previous time period (p < 0.05). In 2016, 13 MenW cases were identified, with an incidence of 0.02 per 100,000 population, four times higher than the average value of 0.005 per 100,000 population observed in the previous years 2000 to 2015.

Among the 63 MenW IMD cases, 53 samples were sent to the NRL for further analyses: 47 bacterial isolates and six CSF or blood samples*.*

### Demographic and clinical data of *Neisseria meningitidis* serogroup W cases

The median age of the 63 MenW cases was 20 years (mean: 29 years), ranging from 1 month to 86 years. Until 2005, MenW was responsible of IMD cases exclusively among children younger than 10 years (the median age was 1 year), except for one. In the period from 2006 to 2016, the median age increased to 26 years.

The female:male ratio was 28:35. Meningitis (25 cases) and septicaemia (22 cases) were the main clinical presentations, followed by meningitis plus septicaemia (16 cases). Four cases had an atypical clinical presentation: two (aged 3 and 26 years) had arthritis; one (20 years-old) had a pericolic abscess and one (5 months-old) had dysentery. Six patients (aged between 22 and 63 years) died, defining a case fatality rate of 10%.

Eleven patients came from foreign countries: Eritrea (n = 1) [23], Mali (n = 1) [23], Ivory Coast (n = 1), Morocco (n = 1) [23], Niger (n = 1), Nigeria (n = 5) and Somalia (n = 1). 

### Microbiological and molecular analyses

#### Antimicrobial susceptibility

Of the 47 MenW bacterial isolates received at the NRL, 44 could be cultured and tested for antimicrobial susceptibility. All of them were susceptible to cefotaxime, ceftriaxone, ciprofloxacin and rifampicin. Moreover, 14 showed decreased susceptibility to penicillin G (PenI, 0.064 > MIC ≥ 0.25) with a MIC_50_ and MIC_90_ of 0.064 mg/L and 0.19 mg/L, respectively.

#### MLST and genotypic formula

The molecular characterisation was performed at the NRL for 51 of 53 MenW. Two samples were not suitable for the molecular analyses. MLST identified two main clonal complexes, cc22 (n = 25) and cc11 (n = 23). In addition, two isolates were cc23 and one was cc60.

**Figure 2:**
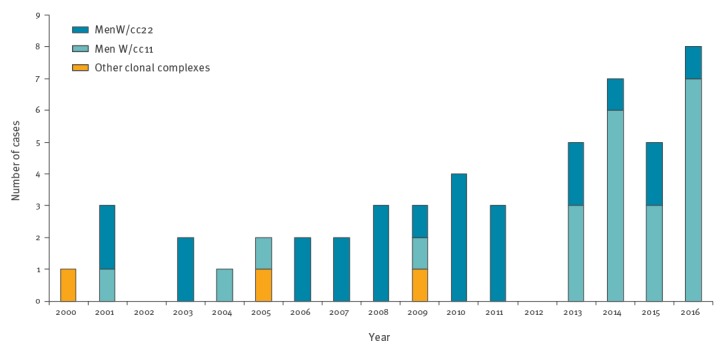
Number of invasive meningococcal disease cases caused by *Neisseria meningitidis,* by clonal complex, Italy, 2000–2016 (n = 51)

As shown in Figure 2, the main clonal complex between 2000 and 2012 was cc22 (19/26); from 2013 onward, the cc11 (19/25) was predominant. Among the 25 cc22 bacterial isolates, eight sequence types (STs) were identified: ST-22, ST-184, ST-3189, ST-904, ST-1286, ST-1959, ST-6779 and ST-11935. Among them, 12 genotypic formulas were reported, of which W:P1.18–1,3:F4–1:ST-22(cc22) was the most frequent (five bacterial isolates; Supplementary Table S1). The 23 MenW/cc11 (18 bacterial isolates and five clinical samples) belonged to ST-11 and presented a single genotypic formula, W:P1.5,2:F1–1:ST-11(cc11) (Supplementary Table S1). The cc23 isolates belonged to ST-23 and ST-9253 and the cc60 isolate to ST-913. 

#### Whole genome sequencing

Whole genome sequencing was performed to identify: the Bexsero antigen sequence types (BAST), the cgMLST, the six antigen-encoding gene profile and the *penA* gene alleles.

##### BAST typing

As shown in the Table, MenW/cc11 clustered in three BAST: BAST 898 (characterised by fHbp peptide variant 1.9, NHBA peptide 96, NadA peptide 6, PorA VR1 5 and PorA VR2 2) for 12 bacterial isolates; BAST 2 (fHbp 2.22, NHBA 29, NadA 6, PorA VR1 5 and PorA VR2 2) for five; BAST 6 (fHbp 2.151, NHBA 29, NadA 6, PorA VR1 5 and PorA VR2 2) for the remaining one.

**Table:** Molecular characterisation of *Neisseria meningitidis* MenW/cc11 bacterial isolates, Italy 2000–2016 (n = 18)

Bacterial isolate ID	ID (http://pubmlst.org/Neisseria)	Year of isolation	BAST	cgMLST sublineage	Six antigen-encoding gene profile
1142	42867	2001	898	Hajj	a
1505	42851	2004	898	Hajj	a
1638	42852	2005	898	Hajj	a
2205	42884	2009	898	Hajj	a
2517	42886	2013	898	Hajj	a
2693	36847	2014	898	Hajj	a
2767	42888	2015	898	Hajj	a
2808	44961	2016	898	Hajj	a
2857	51615	2016	898	Hajj	a
2904	51617	2016	898	Hajj	a
2916	51618	2016	898	Hajj	a
2940	56641	2016	898	Hajj	a
2509	42885	2013	2	South American	b
2593	36848	2014	2	South American	b
2585	36849	2014	2	South American	b
2685	36845	2015	2	South American	b
2858	51616	2016	2	South American	b
2602	42887	2013	6	Singleton	c

BAST: Bexsero antigen sequence typing; cgMLST: core genome multilocus sequence typing; ID: identification code.

##### 4CMenB variant antigens among MenW/cc22

The 4CMenB variant antigens identified among MenW/cc22 isolates were: fHbp peptide variant 2.16, NHBA peptide 20, NadA interrupted by an IS element. The PorA VR1,VR2 were 18–1,3 in eight isolates, 5–1,10–1 in three isolates and 5,2 in one isolate.

##### cgMLST

We included 1,467 of the 1,605 core genome loci in the cgMLST analysis (138 loci incompletely assembled were excluded) for 18 MenW/cc11 and seven reference genomes. 

**Figure 3:**
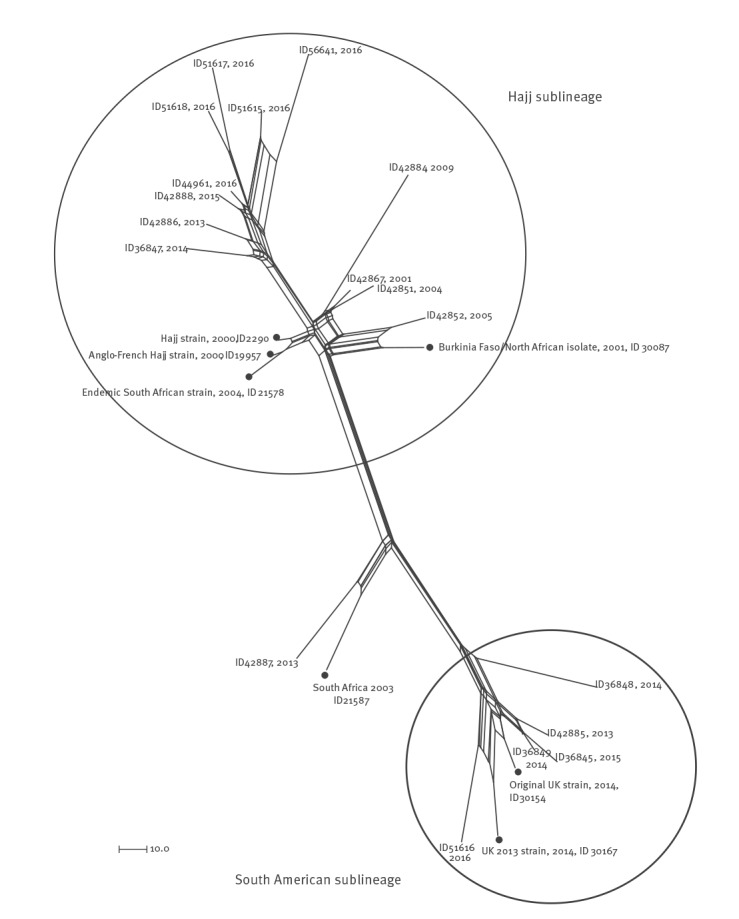
Neighbour-net phylogenetic network based on a comparison of 1,467 core genome loci (cgMLST) of *Neisseria meningitidis* MenW/cc11 genomes Italy 2000–2016 (n = 25)

As shown in Figure 3, the 18 MenW/cc11 split into two main sublineages corresponding to those described by Lucidarme et al. [5]. Twelve genomes (ID 36847, 42851, 42852, 42867, 42884, 42886, 42888, 44961, 51615, 51617, 51618 and 56641) grouped together with reference genomes belonging to the Hajj sublineage, with a mean distance of 89 loci with allelic differences. Eight of these 12 genomes (ID 36847, 42886, 42888, 44961, 51615, 51617, 51618 and 56641) clustered in a subgroup; they had been isolated between 2013 and 2016 and five of them were associated with MenW IMD in patients with Nigerian nationality (Supplementary Table S2).

The figure includes genomes of 18 isolates from this study and seven reference genomes (black dots) available in the *Neisseria* PubMLST website. Last accessed: 10 April 2017. The Hajj sublineage and the South American sublineage are highlighted with circles. For each genome, the ID code from http://pubmlst.org/Neisseria is reported. The scale bar indicates the number of differences between the compared loci.

Our 12 MenW/Hajj sublineage genomes were compared with 128 MenW genomes with the genotypic formula W:P1.5,2:F1–1:ST-11(cc11) and fHbp variant 1.9, identified from IMD cases in Africa (www.neisseria.org; last accessed: 24 November 2017). All genomes showed a mean distance of 60 loci (data not shown).

Five MenW/cc11 genomes (ID 36845, 36848, 36849, 42885 and 51616) clustered together with two genomes in the South American sublineage (ID 30154, as the original UK strain reference, and ID 30167, as the UK 2013 strain reference) [9] with a mean distance of 74 loci. In particular, genomes ID 51616 and ID 30167 showed a higher proximity. The analysis of 27 of 30 genes distinguishing the original and the novel UK strains [9] confirmed that ID 51616 was a UK 2013-strain. The remaining four genomes showed a higher similarity to the original UK strain. The ID 42887 genome was close to the reference ID 21587 (South Africa 2003) in a branch far from both the main sublineages.

For the 12 MenW/cc22 genomes, 1,540 of the 1,605 core genome loci were included in the cgMLST analysis, while the remaining 65 loci were incompletely assembled. The genome comparison highlighted a mean distance of 199 loci (Supplementary Figure S1). The cgMLST analysis of MenW/cc22 and cc11 highlighted high genetic diversity with a mean distance of 588 loci (data not shown).

Overall, the majority of MenW/cc11 were Hajj sublineage (16/22); in particular, it caused five sporadic IMD cases from 2001 to 2013 and 11 cases from 2014 to 2016 (Supplementary Figure S2). Ten MenW/cc11 Hajj were obtained from African refugees and characterised by the presence of fHbp allele 9 (Supplementary Table S2). The South American sublineage appeared in Italy in 2013 and was responsible for five of 22 IMD cases (Supplementary Figure S2). One MenW/cc11 (ID 42887), identified in 2013, did not belong to any sublineage.

##### Six antigen-encoding gene profiles among MenW/cc11

Among the 18 MenW/cc11 bacterial isolates, we found three known profiles [4], comprising the alleles of *porA*, *porB*, *fetA*, *nadA*, *nhba* and *fHbp* genes (Table). Profile a was found in 12 bacterial isolates: 1, 1, 13, 5 (peptide 6), 72 (peptide 96), 9; profile b in five isolates: 1, 244, 13, 5 (peptide 6), 17 (peptide 29), 22; profile c in one isolate: 1, 311, 13, 5 (peptide 6), 17 (peptide 29), 160 (peptide 151).

For four of five clinical samples, we identified *nhba* 72 and *fHbp* 9 (Supplementary Table 2). The remaining sample was not suitable for the analysis.

As shown in the Table, the isolates clustered as Hajj sublineage showed the profile a, the isolates belonging to the South America sublineage showed the profile b and the singleton showed the profile c.

##### *penA* gene characterisation

The 18 MenW/cc11 bacterial isolates were susceptible to penicillin G and showed the *penA* allele 1. Thirteen of 24 of MenW/cc22 were PenI, of which 12 harboured the *penA* allele 14 and the remaining one the *penA* 685.

## Discussion

The epidemiology of IMD is constantly changing. The national vaccination programmes should consider these changes over time and the age groups that are affected most. 

Since 2000, there has been an increase in the number of MenW cases in Europe, America and Africa [2-4]. This international context prompted us to ascertain the current situation of MenW in Italy and how it had evolved over the past 17 years. Although Italy is classified as a country with a low incidence of IMD in the overall population, the number of MenW notified cases has been increasing since 2013. Data collected within the established NSS for IMD reported an increase in MenW cases, even though the absolute number was lower than that reported in other European countries [4-7]. In the past, sporadic MenW IMD cases occurred mainly among children, but have gradually increased also in older age groups, in England since the epidemiological year 2013/14, and in the Netherlands since 2015/16 [7].

In 2016, MenW represented 7% of the total IMD cases reported in Italy. In contrast to other countries [24], very few cases were characterised by atypical clinical presentation; it is likely that this is due to the small total number of reported cases and incomplete available information. In 17 years, cc11 has become the prevalent clonal complex among MenW in the country. In contrast to what was reported in Australia in 2016 [25], MenW/cc11 was not associated with the emerging resistance to penicillin.

The most interesting finding of this study is that both of the MenW/cc11 sublineages, South American and Hajj, cocirculate in Italy. Cocirculation has already been reported in some parts of the African meningitis belt and in South Africa [8], but not in Europe. In the UK in the mid-2000s, the Hajj sublineage was replaced by the South American sublineage [9]. Likewise, in France, the Hajj sublineage, detected up to 2012 [12], was replaced in 2013 by the South American sublineage [26]. The Hajj sublineage appeared in Italy in 2001 and became predominant in 2014. Across the entire study period, the Hajj sublineage represented 73% of the MenW/cc11 identified in Italy. 

Five of the 22 MenW/cc11 were South American sublineage. They appeared in Italy for the first time in 2013, causing five IMD among Italian patients. Four of them were the original UK strain and only one, in 2016, was the UK 2013 strain. As extensively described, the UK 2013 strain has been spreading in northern European countries since 2013 [6,7,9].

In Italy, the National Immunisation Plan 2017–2019 recommends the quadrivalent meningococcal vaccine for adolescents, as the main group of people affected by serogroups Y and W, acting as catch-up or booster of the primary immunisation [27]. The immunisation is also recommended for travellers to countries endemic for the serogroups contained in the vaccine and for people at high risk of IMD [27]. Moreover, the recommendation for the meningococcal B vaccine (4CMenB) for infants before the age of 13 months is administered free of charge. Possible cross-protection against other non-B meningococci, through the presence of the same subcapsular vaccine antigens, need to be evaluated [28,29]. In the UK, serum bactericidal antibody (SBA) activity, promoted by immunisation with 4CMenB vaccine against *N. meningitidis* W strains, was clearly demonstrated [28]. Here, all MenW/cc11 meningococci showed the NadA peptide 6, belonging to the variant 2/3, predicted to be cross-protective with the 4CMenB NadA variant [28]. Moreover, the MenW/cc11/Hajj sublineage isolates showed the fHbp variant 1, one of the antigens of the 4CMenB vaccine. The multi-antigen typing system [30] together with SBA test [31] could define precisely the vaccine coverage against MenW; further evaluations are needed to precisely answer this question also for the MenW identified in Italy.

## Conclusion

In Italy, we observed cocirculation of two sublineages, the Hajj and the South American. This is uncommon and not reported in other European countries. It is likely that the geographical location of our country may favour a peculiar epidemiological situation that needs to be carefully monitored and evaluated.

## Supplementary Data

288000 Supplementary Figure S1

## Supplementary Data

261000 Supplementary Figure S2

## Supplementary Data

147000 Supplementary Table S1

## Supplementary Data

110000 Supplementary Table S2
