# Supplementary material for: Cocirculation of Hajj and non-Hajj strains among serogroup W meningococci in Italy, 2000 to 2016
Source: Euro Surveill. 2019 Jan 24;24(4):1800183. doi: 10.2807/1560-7917.ES.2019.24.4.1800183 (PMC6352001; doi:10.2807/1560-7917.ES.2019.24.4.1800183)
Supplement: Supplementary Figure S2 [file 1800183_STEFANELLI_Supplementary_Figure_S2.pdf]

This supplementary material is hosted by *Eurosurveillance* as supporting information alongside the article "Cocirculation of Hajj and non-Hajj strains among serogroup W meningococci in Italy" on behalf of the authors who remain responsible for the accuracy and appropriateness of the content. The same standards for ethics, copyright, attributions and permissions as for the article apply. *Eurosurveillance* is not responsible for the maintenance of any links or email addresses provided therein.

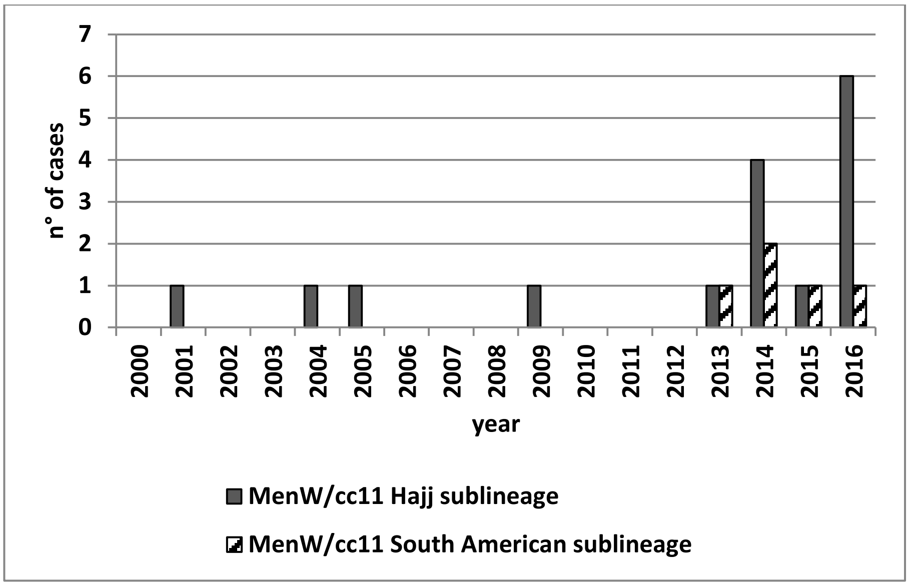

Supplementary Figure 2. Number of invasive meningococcal disease cases due to MenW/cc11/Hajj sublineage and MenW/cc11/South American sublineage by year, 2000-2016.
