# Supplementary material for: Cocirculation of Hajj and non-Hajj strains among serogroup W meningococci in Italy, 2000 to 2016
Source: Euro Surveill. 2019 Jan 24;24(4):1800183. doi: 10.2807/1560-7917.ES.2019.24.4.1800183 (PMC6352001; doi:10.2807/1560-7917.ES.2019.24.4.1800183)
Supplement: Supplementary Table S2 [file 1800183_STEFANELLI_Supplementary_Table_S2.pdf]

This supplementary material is hosted by *Eurosurveillance* as supporting information alongside the article "Cocirculation of Hajj and non-Hajj strains among serogroup W meningococci in Italy" on behalf of the authors who remain responsible for the accuracy and appropriateness of the content. The same standards for ethics, copyright, attributions and permissions as for the article apply. *Eurosurveillance* is not responsible for the maintenance of any links or email addresses provided therein.

	**Year of isolation**	**ID (http://pubmlst.org/Neisseria)**	**Nationality (Age in years)**	**Six antigen-encoding genes profiles**	**fHbp allele**
**Isolate ID**					
2517	2013	42886	Somali (14)	a	9
2767	2015	42888	Nigerian (20)	a	9
2808	2016	44961	Nigerian (26)	a	9
2857	2016	51615	Nigerian (2)	a	9
2916	2016	51618	Nigerian (23)	a	9
2940	2016	56641	Nigerian (0)	a	9
**Clinical samples ID**					
2573	2014	Na	Eritrean (20)	Na	9
2574	2014	Na	Malian (18)	Na	9
2595	2014	Na	Moroccan (37)	Na	9
2854	2016	Na	Nigerien (19)	Na	9

Supplementary Table 2. Characteristics of 10 MenW/cc11 isolates and clinical samples collected from African patients.
